# The Treatment of Disease by Baths and Waters

**Published:** 1900-04-07

**Authors:** 


### The Treatment of Disease by Baths and Waters.

[In commencing a new volume of The Hospital, and in offering to our readers our Special Spring Number, we have thought that it might be well to occupy this and a few of the following pages by something different from the clinical lectures which are usually to be found here week by week; and bearing in mind that this is the opening of the summer volume, it has seemed to us that an article drawing attention to a line of treatment which more particularly belongs to the summer season, might be welcome to our readers. We hope, then, that the following comment on the facts known and on some of the theories held regarding the treatment of disease by means of baths and mineral waters may be found useful and possibly not entirely uninteresting.—Ed. *T. H.*]

### Introductory.

The causes of disease may be roughly divided into those that arise within the patient himself and those that are due to his surroundings; and, in the same way we may divide our means of cure into those which aim directly at modifying the diseased condition into which the patient's body, or maybe his mind, has fallen, and those which aim at altering the surroundings in which he lives, and modifying those very influences which have no doubt been largely concerned in bringing about his illness. It is obvious enough that in the treatment of acute disease both these means of cure are brought into play. Whatever may be a man's daily work, and however his usual surroundings may tend either to health or to disease, all is changed as soon as he is attacked with an acute illness. His work is thrown on one side, his habits are altered, his exercise is cut down; he is kept in a room of one temperature, and put to bed; he is fed on simple slops at stated intervals; he becomes a teetotaller; and, instead of the constant worry of letters and telegrams, he lies at peace. The change produced by such a modification of a man's habits and surroundings is immense, and is often in itself far greater than what is caused by the medicine which he takes. But when we turn to the treatment of those less acute ailments which do not actually lay a man low, then we see how straitened are the opportunities of the physician when he is tied down to physic alone and cannot modify the patient's mode of life. Too often, indeed, the very things that have produced the malady remain in full activity, and we beat helplessly with our pills and tabloids and other medicaments against the strongly-entrenched habits of life which have themselves caused the ailments for which the patient asks relief.

While, then, we recognise to the full that baths and waters have their influence; that the saline constituents of certain waters are really important medicines having effects which require careful study; and that different waters have different actions on the body; and although we admit, what certainly is true, that various hydropathic methods are carried out with very different degrees of effectiveness at different spas, many of which, indeed, have their own specialties which are only feebly imitated at other places; and while we thus agree fully that it is by no means a matter of indifference where a patient is sent; it would, on the other hand, be foolish to shut our eyes to the fact that, complicating, modifying, and, in some cases, over-riding every other influence which goes to make up the spa treatment of disease, is the removal of those home influences which have conduced to its production.

On the understanding, then, that the efficacy of spa treatment by no means depends upon the waters alone, whether they are used for drinking or for bathing, or for both together, it will be well to consider the effects —first, of the external, and, secondly, of the internal use of the various mineral waters.

### Baths and Douches.

The external application of water affects the frame in four different ways—(1) By removing dirt and accumulated secretions, and in regard to the latter by removing them, not only from the free surface, but from the recesses of the glandular structures which lie beneath; (2) by the abstraction or addition of heat, and by the changes set up not only locally but throughout the whole organisms in response to this disturbance of the balance between heat production and heat loss; (3) by "stimulation" of the nerves of the skin; and (4) but only to a very minute extent by absorption of materials contained in the waters applied. The "thermic" and the "stimulating" are by far the most important effects of the external application, for, although certain specific effects are often claimed for certain baths by virtue of their chemical constitution, it may be doubted whether such claims are capable of proof. That certain spas are specially useful in certain diseases is perfectly true; but, as already insisted on, the word "spa" covers much besides bathing in the waters.

By dint of wearing clothes, varying according to the season, man has contrived to do what in the case of most animals is done for them by their hairy covering; that is, he has managed to ensure that the skin shall be kept at a fairly uniform temperature. This, as a rule, runs about ten degrees below the temperature of the blood. As a result, then, of the constant habit of keeping the air which lies in contact with the skin at about 90 Fahr., it happens that a bath at a much higher temperature feels hot, while one at a much lower temperature feels cold, producing in each case a stimulating effect upon the nerves of the skin. But besides this effect such baths have a distinct influence upon the metabolism of the body, the warm ones preventing the escape of heat from, even if they do not actually add heat to the body, while the cold ones, by abstracting heat, necessitate a more active metabolism to keep the internal temperature at its normal level. Even purely local baths have a very wide influence in consequence of the disturbance of the general thermic balance which they produce; but it is in general baths that the effects on metabolism are most strikingly seen.

The first line of defence by which the body protects itself from the abstraction of heat from its surface is by a smart contraction of the vessels of the skin, so that whole surface becomes white and practically bloodless. A great and sudden alteration of the area through which the circulation is distributed is thus produced by the diversion of blood from the skin to the internal organs. But what is termed "reaction" soon takes place, the skin reddens and glows from the large amount of blood now circulating through it, and the greater heat loss from the surface is met by a greater heat production within. This reaction in the young and vigorous occurs quickly and very thoroughly, the effect of the increased metabolism being shown by improved appetite, increased excretions, and a general sense of well-being. But in the old and feeble very different results may follow the application of cold. The vitality may not be sufficient to produce heat enough to meet the loss, reaction may not occur, the patient being left cold and shivery for a long time after his bath; or, even if it does take place, it may be at the expense of great exhaustion; while again, when people are elderly and their blood vessels are rigid and brittle, it may be quite impossible for them to accommodate themselves to such a rapid oscillation, and serious damage may be done to the circulatory system. Cold baths, then, if so arranged as to abstract much heat, as is the case whenever the body is kept submerged for any time, are only fit for the young and vigorous, and their applicability must generally be measured by the readiness with which reaction is established. It should, however, be remembered that, besides abstracting heat, the application of cold to the body is strongly stimulating to the nerves of the skin, and that by means of a sponge down, or a douche, or even by a sudden plunge, followed by friction with hot towels, this good effect can often be obtained without much loss of heat, so that by varying the mode in which cold is applied its range of usefulness is much increased.

Warm baths, on the other hand, so far as their action on the nerves is concerned, are soothing to pain and irritability. Their first effect is usually to very largely increase the vascularity of the skin, and thus relieve internal congestion. Their effect on metabolism is also in large degree the opposite of the effects of cold baths. Instead of involving the necessity of greater heat production, they bring it about that even the heat resulting from the necessary tissue changes is difficult to get rid of, the only exit left being through the lungs. If, then, a hot bath is much prolonged, although metabolism may be at its lowest, both circulation and respiration are greatly quickened.

While, however, cold is rarely applied except by means of water, heat may take many forms, hot air baths being one of the favourite methods of its application; and in regard to these it is necessary to remember that the effect of hot air is very different from that of hot water, chiefly from the very free perspiration which it may be made to produce. No doubt people do perspire even in a water bath, but not nearly to the extent that they can be made to do when exposed to hot vapour or hot air, so that there is this great difference between the two, that, while hot water baths tend to stimulate, and thus to throw increased work upon internal organs, such as the lungs, hot air baths bring into much more full activity not only the whole surface of the skin, but all the glands which lie beneath.

Now, if we turn to the nervous influence of baths, and consider the vast number of sensory nerves which have their termination in the skin, and the enormous number of nerve endings which can be stimulated by exposing the surface to heat or cold, or to the various altered sensations set up by mud or pine or effervescing baths, remembering always that the stimulation of a sensory nerve almost of necessity sets up some form of reflex action, stimulating perhaps the heart, or the vessels, or the secretory glands to varying activity, we can understand that the operation of baths is not a mere question of adding to or of taking away from the sum of the heat within the body. Still, although we have to admit that hydrotherapeutics may be made to play a most important part in the treatment of disease, and although we recognise the great variety of effects which may be produced on the body by different sorts of baths, we have also to admit that these effects are not produced by the absorption into the system of the salts which the waters contain. By soaking into the epidermis, saline and gaseous matters may set up in the living dermis a variety of reactions, and in this way stimulate the nerve endings in different degrees, perhaps in different modes, but there seems no proof at all that mineral-water baths act medicinally by way of absorption of their saline constituents.

We thus see that baths (including air and vapour baths) may be either stimulating or sedative; that they may be made either to increase or diminish metabolism; that they may be made either to increase or diminish the amount of work thrown upon the internal organs; that they may be made to increase excretion by the skin to an extraordinary degree; that when applied locally they may greatly increase the circulation of blood through the part; that, whether hot or cold, local or general, or in the form of air, or vapour, or water, they are great stimulants to the circulation and often to the respiration; and that in many cases the propriety of using them will depend in large degree upon the condition of the vascular and excretory systems.

### The Internal Use of Mineral Waters.

When we turn from the external to the internal use of mineral waters, we see that the conditions on which their activity depends are to a certain extent reversed, their therapeutic effects being in this case largely influenced by their chemical constitution. First of all about their one common constituent—water. There can be but little doubt that much of the effect produced at many spas is due to the water taken, and that the saline constituents, although making this more digestible, have often but little part in its curative effects. Such water drinking tends to increase the secretions; for a time it increases the amount of urea, and possibly of other matters, discharged, and, speaking in popular terms, which, in this case would not be very far from the truth, it may be said to wash out the tissues. It is certain that a very large proportion of the water which is drunk is absorbed and enters into the blood stream, and that before it is excreted by the kidneys it has many opportunities of materially modifying tissue metabolism. In fact, the diuretic action which follows the drinking of large quantities of water, an action which is among the most constant of the effects of spa treatment, is but the result of an alteration of the composition of the blood which is felt by all other organs equally with the kidneys, and there is every reason to believe that the same influence which, by acting on the glomeruli of the kidney, increases the outflow of urine, when acting on the cells of other organs sets up a corresponding increase of the osmotic interchange between blood and tissue which forms one mode, at least, by which the cells are fed, their function is maintained, and their waste material is removed. Markedly is this the case in regard to the stomach and the intestines, and, perhaps, even more marked in regard to the liver, through which the diluted blood from these absorptive surfaces is first carried, and it may be broadly stated that what is seen as diuresis is but an outward sign of a process which has gone on in some degree in relation to every cell in the body, each of which has first absorbed a little more, and then has lost a little more fluid, than it would have done if the normal amount of liquid taken at one time had never been exceeded. We can hardly doubt that with this greater interchange of fluid between blood and tissue a greater interchange of solids takes place also, and, indeed, that this is so is shown by the greater excretion of urea, which, for a time, at any rate, follows increased water drinking. Thus we have truly a washing-out of the tissues, and a disturbance of much effete material which would have "lodged," as the saying is, so long as the ingestion of fluid remained steadily at one normal and unvarying standard. To whatever spa we go and whatever mineral water we drink, it will be seen that this is one main element in the success of the treatment, and it is not altogether impossible that, in regard to some of the waters of which we shall have to speak, the saline constituents with which they are charged act rather by way of rendering them digestible and easily absorbed than by any specific action which they may be supposed to have upon the tissues of the body.

### Classification of Mineral Waters.

In the classification of mineral waters we cannot do better than follow that which is given in Dr. Hermann Weber's admirable work on "The Mineral Waters and Health Resorts of Europe." Mineral waters, then, may be considered under the following heads: (1) Simple or indifferent waters. (2) Common salt or muriated waters. (3) Alkaline waters. (4) Sulphated and muriated sulphated waters. (5) Iron or chalybeate waters. (6) Arsenical waters. (7) Sulphur waters. (8) Earthy or calcareous waters. (9) Table waters and other very weakly mineralised cold waters.

### The Simple or Indifferent Thermal Waters.

Many of these waters have a high repute, notwithstanding their very slight mineralisation, but it is to be noted that at many of the spas where they are to be found very complete arrangements also exist for douching and massage, and for carrying out those accessory forms of treatment on which success so largely depends. Still, even granting the efficacy of fresh air and change of scene, and of all those modern methods of manipulations which are now used in addition to the mere simple baths, it is a very curious thing, and one very difficult of explanation, that such striking results as are found to occur should follow the use of indifferent waters, such, for example, as those of Buxton, even when, as is often the case, nothing is done beyond the internal and external employment of the waters. It is probable that the simple thermal waters act principally, as we have already said, by washing out the tissues. The cases of chronic gout and rheumatism, the chronic skin diseases, the old inflammatory effusions in the pelvis, the abdomen, or around wounds and cicatrices, and the cases of neuralgia, peripheral neuritis, and nerve degeneration, which flock to these spas, and undoubtedly receive benefit from their visits, are often due to gouty and rheumatic conditions, and other forms of auto-intoxication which are best relieved by increased elimination, such as is caused by drinking these waters, and perhaps even more by the effect on the metabolism set up by immersion in water, which in most cases is somewhat below the body temperature, especially when these are combined with residence in a pure air in a bracing locality.

*Some Simple Thermal Waters, with Their Temperature in Degrees Fahrenheit.*

Bath (104 deg. to 120 deg.)
Buxton (82 deg.)
Matlock Bath (68 deg.)
Wildbad (91·5 deg. to 104·5 deg.)
Ragatz-Pfaefers (89 deg. to 98·5 deg.)
Chandefontaine (96 deg.)
Schlangenbad (81·5 deg. to 89 deg.)
Badenweiler (79 deg.)
Gastein (both tepid and hot).
Teplitz (83 deg. to 114 deg.)
Leukerbad (102 deg. to 124 deg.) A calcareous water. Specialty, prolonged immersion baths.
Saint Amand. Chiefly known for its mud baths, which really are allied to sulphur baths.
Plombières (a wide range of temperature).

### Muriated or Common Salt Waters.

Passing now from the consideration of the effects of what must always be the fundamental basis of hydrotherapeutics, namely, the use of simple water, let us consider to what extent the effects so produced are modified, and what new effects are super-added, when the water is so charged with various salts or gases as to be not merely a spring water but a truly mineral water, one "impregnated with mineral matter," as the dictionary has it. Before going further, however, we must point out that the composition of all mineral waters is very complex, and that each contains many different salts, so that although they may be classified as muriated, sulphated, alkaline, &c., as the case may be, such a classification is founded merely upon the predominance of one or other constituent in their composition, and is by no means intended to suggest that their active principles are exclusively of the nature indicated by the name of the group in which they are placed. Indeed, if the salt, the predominance of which in certain waters leads them to be classed together in one group, were removed, the different members of such a group might be scattered and placed under quite different categories in consequence of the widely varying characters of the constituents remaining in each.

In regard to many spas, indeed, it is a matter of considerable doubt under which head they should be placed. Although, then, for purposes of study and discussion it is necessary to make use of a classification depending on predominant constituents, the effects of the other substances which the different waters contain in varying proportions, and the influence of the different surroundings at different spas, and of the special facilities for treatment existing at certain places, must never be lost sight of, for it is on these that often depend the peculiar virtues of the individual spas in the treatment of particular classes of disease.

Coming back then to our text, "the muriated waters," we find that, although they contain many things besides common salt, this is in each case their main constituent, and thus we have to consider in what way does the addition of this saline matter modify the effect of the water.

First, in regard to external use we do not go far before we see plainly how much more stimulating salt water is than plain. This stimulating effect is produced in two ways, partly by the direct stimulation of the nerve endings, and partly by the irritation of the tissues. When the epidermis becomes soaked with water every nerve-ending which lies buried beneath is titilated by a new sensation, and the sum of these sensations, as felt through the thousands of nerve-endings spread over the whole body, becomes a very potent stimulus to the nervous centres. We may be quite sure that such a torrent of sensations as are thus poured inwards through the conducting fibres are not lost, but produce some effect besides merely being felt by the sensorium. How much greater then is this effect when the water with which the skin is soaked is of an irritating nature in consequence of being charged with salt. Of this we are at once conscious when we bathe in sea water, a proceeding the superior bracing and stimulating effect of which, as compared with bathing in a river, even although the water may not be nearly so cold, is perfectly well recognised. But salt water also produces a more or less abiding irritation of the tissues of the dermis, setting up a vascular congestion which continues for a considerable time, in some instances giving rise to uticaria, or even going so far as to produce patches of inflammation commonly spoken of as eczema. The tissue irritation so caused again reacts upon the nerves of the skin, so that in every way salt baths become more "stimulating" than those provided by the simple thermal waters. So obvious is this effect that at many spas arrangements are made to vary the activity of the baths in both directions, sometimes diluting them, but more often enforcing their activity by adding various proportions either of strong salt solutions or of the mother liquors left after the crystallisation of the salt the waters have contained. It should be noted, moreover, that the addition of these "mother liquors" does not merely increase the strength of the waters, although it does this to a considerable extent. In most cases the mother liquors contain far more than their due share of the salts other than sodium chloride which the original water had contained, and especially of the very deliquescent calcium chloride, which, no doubt, has a special influence of its own upon the skin.

Many of the foreign muriated waters also contain a considerable amount of free carbonic acid gas, which, escaping in effervescence all over the surface of the patient as he lies immersed in the bath, adds to its effects a mechanical stimulation which is quite peculiar, and seems to produce results which cannot be obtained in any other way. This is one of the special peculiarities of the baths of Nauheim, so celebrated for their utility in certain heart affections, the baths there consisting of a strong muriated water constantly effervescing with carbonic acid gas, the effect in some cases being increased by agitation of the water as in the wave or surf bath. The internal use of the muriated waters is more stimulating to the mucous membranes, to the gastric glands, and the liver cells than that of plain water. The saline is also often more easily digested than the simple water, and when it contains carbonic acid the evolution of this gas adds very considerably to the peristalsis of intestines which is set up by its ingestion. These waters are laxative rather than purgative, but in this respect they vary greatly in different individuals, and they are generally diuretic. They also have a not inconsiderable influence on the secretion of all the mucous membranes, and thus prove serviceable in many kinds of chronic catarrh of various organs. But it is not only by their action on the mucous surfaces that they prove beneficial. In a much greater degree than plain water do muriated waters act as stimulants to metabolism, with the result that the activity of tissue change is much increased by their use. It is probable also that, to certain extent, the use of these waters increases the capacity for absorbing and digesting nourishment; for, although it is clear that much of the alteration of body weight which is sometimes produced during a course is due to the diet prescribed, it is the general experience that independently of spa treatment it is much more difficult to produce such effects, so that we may presume that in the presence of the increased tissue change induced by muriated waters, patients become more susceptible to the influence of dietetic treatment than is the case under ordinary circumstances. We must not omit to point out that in many cases waters which are spoken of as "muriated" contain also other ingredients which have their own distinct effects. Many of them, for example, contain iron, some iodine, or bromine, or arsenic, and on these things often depends a special efficacy of particular spas in particular diseases.

*Some Muriated or Common Salt Waters.*

Seaside resorts may be included in this category. They are very useful in cases of scrofula and rickets, and, where hot sea-water baths are provided, they are useful in various forms of chronic rheumatism and gout.

Droitwich (saturated) baths are used in chronic rheumatic and gouty affections, and also in carrying out the "Nauheim treatment" in heart cases

Woodhall Spa (also bromides and iodides).

Sidmouth, where sea water is used for the "Nauheim treatment."

Nantwich.

Llangammarch Wells (contains also chlorides of barium, calcium, and magnesium). Makes a specialty of heart cases.

Bridge of Allan.

Nauheim (some iron, much carbonic acid). Useful in many scrofulous and catarrhal conditions, rheumatism, and neuralgia, but of late years has become "special" for heart disease.

Oeynhausen (highly gaseous). So far as the baths are concerned is very like Nauheim, but not so warm until artificially heated.

Rheinfelden.

Kreuznach (minute traces of strontium, barium, and arsenic, with sodium bromide and iodide). Special for scrofula and rickets, catarrhs, and chronic inflammatory gynæcological cases. Inhalations used.

Homburg (a little iron and a dash of sulphur). Gout, uric acid diathesis, chronic rheumatism, and chronic female complaints.

Wiesbaden. Chronic gout and rheumatism, syphilis, inflammatory female complaints, and catarrh of the upper air passages.

Kissingen.

We now come to the consideration of a series of waters the taking of which, much more than in the case of the simple or the muriated waters, may be compared to the taking of physic. These are the alkaline the sulphated, the iron, the arsenical, the sulphur, and some of the earthy waters.

### Simple Alkaline Waters.

The predominating constituent in these waters is sodium bicarbonate, to which are added in some cases iron, lithium, &c. As in the ordinary drug treatment of disease alkalies have a large field of utility, but just as in prescribing these drugs in the consulting-room, so at the spas, it does not generally do to continue their use for very long at a time, partly because of their reducing effects and partly because, after their use has been continued for a time, their efficacy seems to fail, tonics, or in bath parlance, "after-cures," being required to complete the treatment. They soothe gastric irritability, or, in other words, acidity, and just as in the soda mixtures so commonly prescribed they increase the flow of gastric juice. Hence it comes about that they are very largely used in the treatment of various kinds of dyspepsia, and in all forms of catarrh of the stomach and intestines.

Again, just as every practitioner puts sodium bicarbonate in his cough mixtures because it helps to "loosen" the expectoration, so one sends cases of bronchial catarrh and other cases whose mucous secretions are tough and viscid to drink the alkaline waters, simple or muriated.

Probably it is much in the same way by lessening the viscidity of secretions that these waters act so beneficially as they do in cases of portal congestion, of gall-stones, and of "torpid liver." Many forms of gout also seem to be relieved by their use, notwithstanding the objection which is often raised to the taking of soda compounds when the blood is charged with uric acid products. Alkaline waters also act pretty strongly as diuretics, and one of the effects which they produce, one on which patients are apt to lay much stress, is loss of flesh. As a part of this tendency to produce absorption of unused tissue is one of their secondary results, namely, their tendency to promote the absorption of inflammatory products, such, for example, as remain after old pelvic disorders in women.

But, indeed, the variety of morbid conditions for which alkaline waters may sometimes give relief is very considerable. If any medical man will consider for a moment in how many cases a day he finds it desirable to order alkalies, and in how many cases of catarrh of every organ, of torpid liver, of slow digestion, of inactive kidneys, of gout, and of those many stiff joints and painful limbs which go by the convenient name of rheumatism, he finds it good to order the old familiar "Sodæ Bicarb," the very drug which forms the staple of alkaline mineral waters, he will recognise at once how wide is the field for their use, and how popular they are among the patients whom they suit.

Alkaline baths are often ordered, but it is probable that, except for their greater cleaning properties, their action does not differ very materially from simple thermal baths at corresponding temperatures and equivalent gaseation.

*Some Simple Alkaline Waters.*

Vichy (hot). Vals (cold). Neuenahr. Obersalzbrunn. Bilin.

### Muriated Alkaline Waters.

Among the effects of the simple alkaline waters is diuresis, by which a large quantity of chloride of sodium is removed from the body. Thus it happens that in some cases it is better to use an alkaline water which contains enough of the latter salt to replace the loss. But in other ways the effect of the muriated is somewhat different from that of the simple alkaline water. It seems to be less depressing, it does not cause so much loss of flesh, and it is found by some that it has not that tendency to bring on an attack of gout which is often noted to be the case at the simple alkaline spas. The muriated alkaline waters also have a somewhat special action in various catarrhal conditions, such as cervical leucorrhœa, and where these waters are combined with iron or with arsenic they are found very useful in cases of anæmia and cachexia.

*Some Muriated Alkaline Waters.*

Ems. (Highly gaseous, also a little iron in some.) Pulverised water for inhalations.

Royat. (Iron waters.)

Assmannshausen. (Contains some lithium).

### Sulphated Alkaline Waters.

The addition of sulphate of sodium to an alkaline water clearly alters considerably its mode of action. Besides a much higher degree of stimulation of the liver and direct chologogue effect, purgation comes into play, and the waters thus become much more active in their depletive effects upon the abdominal viscera, the tendency of sodium sulphate being to produce free watery evacuations. Thus they are used in the treatment of disorders which arise from free living, and by people who wish to diminish their load of fat. The treatment of obesity is a strong point at the spas with sulphated alkaline waters. Cases of gastric and intestinal catarrh resulting from hepatic disorder, hæmorrhoids, congestive biliary derangements arising from high feeding, cases of catarrhal jaundice, hepatic glycosuria, and various forms of enlargement of the liver, as well as of gall-stone, all find relief from these waters if they can stand them. Habitual constipation is also well treated by such waters. The constipation is overcome for the time by the purgative action of the waters, but at the same time habits as to diet and exercise are inculcated and practised, which, if kept up, have a permanent influence on the morbid condition. The same may be said of the cases of uric acid diathesis in stout people, which flock in such crowds to these waters. They derive great benefit from the waters, as, indeed, many of them have done again and again before from the domestic use of such stronger cathartics as Friedrichsall or Hunjadi water; but at the same time that they are being relieved by the direct medicinal action of the waters the morbid habit of body on which their disease depends is being attacked in other ways which it would be hopeless to undertake at home.

There are many cases also of recurrent headaches connected with abdominal plethora—what Dr. Haig would call uric acid headaches—which are very beneficially influenced by waters of this character. In conjunction with the drinking of these waters baths are employed in some cases, but whether the water has any specific effect may perhaps be doubted. In many cases, however, in which the internal use of these waters is beneficial the use of baths is also of much service, especially when, as in the Turkish bath, they are made to produce free diaphoresis. At all the sulphated alkaline spas many of the patients are full-blooded, stout people who have indulged freely at the table and not taken enough exercise, and who suffer in consequence from dyspepsia, constipation, and various degrees of auto-intoxication, often with enlarged livers and flabby hearts. But where, as at Franzensbad, the water also contains iron, we find the same chlorotic and anæmic clientèle as is generally to be met with at the chalybeate spas. Women with pelvic difficulties also visit these baths, and often receive much benefit; the rest and the self-indulgence which the existence of uterine disorders so often induce, leading to very much the same condition of the viscera as is met with among alcoholic City men, a condition to be relieved by purgative alkaline waters, by regulated diet, and by moderate exercise and early hours.

*Some Sulphated Alkaline Waters.*

Karlsbad. Marienbad. Franzensbad. Tarasp.

Sulphated Waters and Muriated Sulphated Waters.

The first of these are strong aperient waters mostly used bottled. For many "congestive" liver cases they are of great advantage, their action on the liver and, secondarily, on the urine, by virtue of the sodium sulphate which some of them contain in considerable quantity, being often very useful. The chief objection to their home use is that by relieving symptoms they rather tempt people to continue the mode of life which has led to their disorders. Amongst these waters may be mentioned Hunyadi-Janos, Æsculap, Apenta, Püllna, Friedrichshall, and the very strong Rubinat waters. The muriated sulphated waters contain principally chloride of sodium, together with some sulphate of sodium and magnesium.

*Some Muriated Sulphated Waters.*

Cheltenham. Leamington. Brides-Salins. Saint-Gervais.

Chalybeate Waters.

The effect of iron taken as it occurs in mineral waters is to all intents and purposes the same as when it is taken as a medicine, with these reservations, however, that many patients who are upset at once by iron in ordinary form can take it perfectly in chalybeate waters, that chalybeate waters sometimes contain other substances, such as alkalies and salines and carbonic acid, by which the effect of the iron is much enhanced, and that most people who require iron are also eminently fitted to receive benefit from the other adjuncts — the regulated diet, the early hours, the open air life—which go to make up spa treatment. In fact, the very word spa is derived from the name of one of the best known chalybeates. No doubt only very little of the total quantity of the iron swallowed is absorbed, and just the same may be said of the steel drops and ferruginous tonics taken at home, but that the iron does increase the number of the red corpuscles, and of the amount of hæmoglobin which they contain, seems clear, although the exact manner in which the non-living is turned into the living iron may be as open to question at the spas as in the out-patient room. But this much seems clear, that, as we have said, in speaking of the use of simple springs, the drinking of considerable quantities of water has an important influence upon the osmotic circulation within the tissues, and there seems every reason to suppose that these tissues have a far better opportunity of seizing a substance of which they are in want at a time when this "in and out" circulation is going on with considerable activity, than when its operation is limited to the mere renewal of the waste of the tissues concerned.

The cases treated at chalybeate spas include chlorosis and all kinds of anæmia, slow convalescence from various diseases, cases of dyspepsia with debility, and a crowd of disorders of the female generative organs which, either as cause or as effect, are associated with anæmic states. Baths and douches of various kinds are largely used. The baths which are so commonly employed at the chalybeate spas, especially those which contain a large amount of carbonic acid, are extremely useful, and greatly add to the efficacy of the treatment, although not perhaps as a direct consequence of the iron they contain.

*Some Chalybeate Waters.*

Tunbridge Wells. Cheltenham. Harrogate. Spa. Schwalbach.

Pyrmont (gaseous and muriated), a form of mud bath, is made from a ferruginous peat met with in the district.

St. Moritz (well gaseated, but weak in iron). Great climatic advantages in the treatment of chlorosis.

Arsenical Waters.

The amount of arsenic contained in any of these waters is but small, and in some cases is quite insignificant; still in the stronger of them the amount is sufficient to produce a real therapeutical influence. The arsenic occurs in association with sulphate of iron, or with bicarbonate of iron, or with muriated alkaline waters. There can be no doubt that the French spas of La Bourboule and Mont Dore are very efficacious.

*Some Arsenical Waters.*

Bath (only slight traces).

La Bourboule (muriated alkaline waters).

Mont Dore (some springs contain a little iron). Asthmatic cases do well.

Levico (also ferruginous).

Vals (also cold alkaline and weak iron sulphate).

At most of the arsenical spas the water is pulverised for inhalation purposes. Mont Dore has a very special reputation for the treatment of cases of chronic laryngitis and catarrhal affections of the respiratory organs.

### Sulphur Waters.

The amount of sulphur or of sulphur salts which some of these waters contain is but small; nevertheless, in whatever part of the world they occur they have gained for themselves a considerable repute as curative agents. The fact has to be recognised that, quite irrespective of any theory as to their action, the "stinking waters" have been regarded as effectual agents in the cure of disease for a long period, and that, so far as mere experience is to be accepted as a guide, waters which evolve sulphuretted hydrogen are to be regarded as beneficial in such cases as the following: Chronic rheumatism, gout, plethora, syphilis, skin diseases, scrofulous diseases of bones, and in many other cases of chronic suppuration. Sulphur as it occurs in sulphur waters is a purgative alterative, but sulphur is by no means the only ingredient in what are called sulphur waters, and much of their efficacy is no doubt due to their saline constituents.

Many sulphur baths are credited with special virtues in the treatment of syphilis, and have been thought to "bring out" the symptoms again, and thus to show when the disease is "scotched but not killed." Be this as it may, the reputation of certain sulphur spas in syphilis has for long stood so high that they have become the centres of a sort of specialty in the treatment of such cases, so that, at such places, for example, as Aachen, Uriage, or Luchon, and in less degree at Harrogate and many other sulphur spas, it is easier to obtain skilled and experienced antisyphilitic treatment than at other watering-places. It must, however, be remembered that the treatment in vogue consists of various modifications of the well-recognised forms of mercurial treatment, and that, although, as everyone knows, bathing is useful, it is chiefly as an adjuvant to more active measures that it is effectual. Still, this has to be said, that the more active the tissue change the more free the diuresis, and the more perfect the action of the skin, the more effectual and the more safe does a mercurial course become; and when to these advantages, which the thermal and the muriated sulphur waters give, we add the facilities for inunctions and medicated vapour baths being given with all proper precautions we may well send syphilitic patients to such special spas.

Strong sulphur waters are distinctly laxative, with some people purgative, and it is generally considered that unless a moderately purgative action is produced some aperient should be taken in addition, so that, in considering the effect of the sulphur waters, it is well always to place them among the aperient waters. They also have a very distinct and special action on the liver, and it seems probable that they also act as tonics to the nervous system.

#### *Some Sulphur Waters.*

Harrogate. (Many springs, most of them cold muriated waters containing sulphuretted hydrogen and sodium sulphide. One also contains barium chloride. Harrogate has also other springs which are not sulphur waters, some containing chloride and others carbonate of iron).

Askern Spa.

Llandrindod Wells.

Strathpeffer (also chalybeate).

Moffat (also chalybeate).

Lisdoonvarna (also chalybeate).

Bagneres-de-Luchon.

Cauterets. Much used in affections of the respiratory organs, especially laryngitis, and in gouty and catarrhal conditions.

Eaux Bonnes. Catarrhal affections.

Aachen. Bronchial, laryngeal, and stomachic catarrh, chronic rheumatoid arthritis, rheumatism and gout, but especially syphilis.

Aix-les-Bains. All forms of chronic gout and rheumatism, neuralgia, stiff joints, chronic skin diseases, and catarrhs. Many cases of syphilis are also treated.

Barèges. Skin diseases. Old wounds and painful cicatrices.

St. Sauveur. Much used in nervous disorders and in female complaints.

Uriage. (A muriated warm sulphur water.) Much used in chronic skin diseases, in diseases of the female pelvis, and in syphilis.

### Earthy or Calcareous Waters.

The mineral matters, on account of the preponderance of which these waters are classed together, are calcium carbonate, calcium sulphate, and magnesium carbonate. When magnesium sulphate occurs in any quantity they fall into the class of "bitter waters." They are mostly diuretic, partly as the result of the earthy salts which they contain, partly in consequence of the amount of water drunk, and partly, perhaps, from the astringent action which in moderate doses they seem to exercise upon the intestinal mucous membrane, by which the whole of the excess of fluid ingested is thrown upon the kidneys, in place of passing off in the form of relaxation of the bowels, as is the case with so many other forms of mineral water.

Earthy waters are of special service in catarrhal and other diseases of the urinary organs, and particularly in cases in which urinary calculi are threatened, or have actually formed in the kidneys or the bladder. But just as Aachen and other sulphur spas have become great resorts for the treatment of syphilis by all sorts of methods, and not by bathing alone, so have some of the earthy springs become special centres for the treatment of urinary diseases—treatment in which the waters do but play a part. Operative interference is, indeed, largely practised, and, although no one can doubt that in many cases the thorough wash-out which the waters give to the urinary passages has a great influence in removing small calculi, it must be recognised that much else besides water drinking goes on at these spas.

### Conclusion.

In such a short comment as has here been given upon the varied action of the different spas and mineral waters, it has been impossible even to indicate in the slightest way the sort of cases which more especially flock to the places mentioned. Lack of space has made it necessary to confine attention strictly to groups of waters, to leave out details, and to omit entirely all reference to the clinical selection of cases for different spas—a topic which might, no doubt, have been handled in a more interesting manner than has been possible in dealing with mere generalities. Perhaps we may on some future occasion be permitted to deal with the clinical aspect of the question. In the meantime the writer of this article desires to acknowledge his indebtedness to many writers on the subject of mineral water, especially to Sir Hermann Weber, Dr. Burney Yeo, Dr. Macpherson, Dr. George Oliver, Dr. Myrtle, to many writers of local treatises, and also to B. Bradshaw's "Dictionary of Health Resorts."

